# Serum Therapy Antedated

**Published:** 1896-10

**Authors:** 


### Correspondence.

### Serum Therapy Antedated.

The JOURNAL is in receipt of the following interesting communication from Dr. Nunez de Villavicences, an intelligent Spanish physician, a Cuban refugee at present sojourning in Gonzales. Dr. Nunez is a general practitioner of large experience, and has been a close observer. He is also extensively read, and takes an interest in every advance in science. He is, moreover, a constant reader of the "Red Back." The doctor writes:

While traveling, in 1881, in the State of Bolivar, U. S. of Columbia, I observed in the town of Mompox, situated on the Magdalena river, a new method of treating the hydrophobia. Serum therapy was practically in vogue at that time.

In the town of Lincee, a few miles from Mompox, in 1878, Dr. C. Arango observed that a mad dog that had been castrated lapped the blood from the wound and was cured. The doctor's attention was called to this fact. In the same town, in the same year, other dogs were mad, and the same operation was performed, and of nine dogs five were saved. The doctor deduced that the blood lapped had cured them. Two horses and three cows were bitten by mad dogs in the same year, 1878, and Dr. Arango gave them two spoonsful of blood (taken from each animal) for several days, and only two cows died.

Dr. Arango continued his observations. In both places, Mompox and Lincee, he treated, in 1879-1880, several cases of hydrophobia by the same method. He commenced similar treatment in the human being in 1879, and up to July, 1881, Dr. Arango had cured fifty-two persons out of sixty bitten, and had saved also a great number of animals, horses, donkeys, cows, pigs, etc.

Dr. Charles Arango waits for eight days to pass, and then commences the method, and continues it for several days.

I was in Mompox in 1881, when a Mrs. Mary Rosary de la Ossa and Mr. Benito Cruz were bitten by mad dogs, and Dr. Arango was called and saved them.

Many other instances have been published (in 1881) in the daily newspaper *Opinion Nacional*, from Caracas, Venezuela.

DR. NUNEZ DE VILLAVICENCES.

